# Elastic power but not driving power is the key promoter of ventilator-induced lung injury in experimental acute respiratory distress syndrome

**DOI:** 10.1186/s13054-020-03011-4

**Published:** 2020-06-03

**Authors:** Patricia R. M. Rocco, Pedro L. Silva, Cynthia S. Samary, Muhammad K. Hayat Syed, John J. Marini

**Affiliations:** 1 Laboratory of Pulmonary Investigation, Carlos Chagas Filho Biophysics Institute, Federal University of Rio de Janeiro, Rio de Janeiro, Brazil; 2 Department of Physiotherapy, Faculty of Medicine, Federal University of Rio de Janeiro, Rio de Janeiro, Brazil; 3 Department of Pulmonary and Critical Care, Regions Hospital, MS11203B, 640 Jackson St., St. Paul, MN 55101 USA; 4 Division of Pulmonary, Allergy and Critical Care Medicine, University of Minnesota, Minneapolis, Minnesota USA

**Keywords:** Ventilatory power, Ventilator-induced lung injury, Acute respiratory distress syndrome, Inflammation, Alveolar collapse

## Abstract

**Background:**

We dissected total power into its primary components to resolve its relative contributions to tissue damage (VILI). We hypothesized that driving power or elastic (dynamic) power offers more precise VILI risk indicators than raw total power. The relative correlations of these three measures of power with VILI-induced histologic changes and injury biomarkers were determined using a rodent model of acute respiratory distress syndrome (ARDS). Herein, we have significantly extended the scope of our previous research.

**Methods:**

Data analyses were performed in male Wistar rats that received endotoxin intratracheally to induce ARDS. After 24 h, they were randomized to 1 h of volume-controlled ventilation with low *V*_T_ = 6 ml/kg and different PEEP levels (3, 5.5, 7.5, 9.5, and 11 cmH_2_O). Applied levels of driving power, dynamic power inclusive of PEEP, and total power were correlated with VILI indicators [lung histology and biological markers associated with inflammation (interleukin-6), alveolar stretch (amphiregulin), and epithelial (club cell protein (CC)-16) and endothelial (intercellular adhesion molecule-1) cell damage in lung tissue].

**Results:**

Driving power was higher at PEEP-11 than other PEEP levels. Dynamic power and total power increased progressively from PEEP-5.5 and PEEP-7.5, respectively, to PEEP-11. Driving power, dynamic power, and total power each correlated with the majority of VILI indicators. However, when correlations were performed from PEEP-3 to PEEP-9.5, no relationships were observed between driving power and VILI indicators, whereas dynamic power and total power remained well correlated with CC-16 expression, alveolar collapse, and lung hyperinflation.

**Conclusions:**

In this mild-moderate ARDS model, dynamic power, not driving power alone, emerged as the key promoter of VILI. Moreover, hazards from driving power were conditioned by the requirement to pass a tidal stress threshold. When estimating VILI hazard from repeated mechanical strains, PEEP must not be disregarded as a major target for modification.

## Background

In current clinical practice, attempts to evade ventilator-induced lung injury (VILI) typically center on avoidance of excessive end-inspiratory static pressure (Pplat) and driving pressure (ΔP). The ΔP, the difference between Pplat and PEEP, is the ratio of tidal volume (*V*_T_) to tidal compliance (C). While these measures are useful, they account neither for dynamic features of inflation [1] known to influence VILI (flow rate and profile) nor for the respiratory rate (RR) with which the lung is exposed to tidal cycling [2]. To rectify this deficiency, considerable data gathered in recent years have turned clinical [3] and investigative attention toward a more conceptually attractive and inclusive variable, total ventilating *power*, the product of tidal energy and respiratory rate [4–7].

Total power is a bedside-measurable index that includes all dynamic and static pressures that influence strain during tidal volume delivery and accounts for the rate at which tidal energy is repeated [2, 8, 9]. Tidal energy delivered to the lung is determined as the product of applied stress (estimated by airway or transpulmonary pressure) and the resulting incremental strain (estimated as tidal volume). Experimentally, the externally measured power necessary to injure the lung depends upon multiple factors. Prominent among these are the transpulmonary pressure, the size of the aerated baby lung, the pulmonary hemodynamics, the metabolic status (temperature, PaCO_2_), and the vulnerability of the tissue to repeated strain [2].

Despite the appeal and mechanistic logic of measuring total power in preference to Pplat and ΔP at the bedside, controversy has arisen regarding several aspects of that proposal. Among these are the following: (1) whether the more easily measured ΔP alone or the product of ΔP and respiratory rate might serve as well as total power (Pwr) to assess VILI risk and (2) whether Pwr or only its *V*_T_ influenced piece, the *driving* power (ΔP × *V*’_E_), is the dominant component influencing parenchymal damage. Moreover, some have argued that power applied to overcome flow resistance and PEEP can be discarded [10], as during each inflation cycle, the former dissipates primarily through the endotracheal tube and central airways proximal to the alveoli at risk, and the latter is temporarily stored as elastic potential energy to be dissipated across the exhalation valve before the next inflation cycle begins [1, 10]. Some recent experimental data, however, indicate that all components of energy and power interact with one another and have the potential to damage when any of them is abnormally high and sufficient time is allowed for VILI expression [6, 11]. This latter observation, however, does not imply that each category of inflation energy is equally potent as an injury stimulus or that the raw total power serves clinically as a reliable and sensitive indicator of VILI risk. Therefore, dissection of total power into its primary pressure components to determine the relative strength of their contributions to tissue damage may be instructive. Along this line, whether driving power or elastic (dynamic) power can be used as an equally valid or more precise and consistent VILI risk indicator than total power is of particular interest. To address this question, we examined the relative correlations of these three measures of power with VILI-induced histologic changes and injury biomarkers as PEEP was varied, using pre-collected data from a rodent model of mild-moderate acute respiratory distress syndrome (ARDS) [12].

## Materials and methods

### Study approval

This study was approved by the Animal Care Committee of the Health Sciences Center, Federal University of Rio de Janeiro (Rio de Janeiro, Brazil). All animals received humane care in compliance with the National Society for Medical Research “Principles of Laboratory Animal Care” and the US National Academy of Sciences “Guide for the Care and Use of Laboratory Animals” (Washington, D.C.).

Experimental data were examined from unanalyzed but pre-collected data from a study of 30 male Wistar rats with acute respiratory distress syndrome that tested the biological impact of transpulmonary driving pressure [12]. Briefly, animals with endotoxin-induced mild-moderate ARDS were mechanically ventilated (Servo-i; MAQUET, Sweden) at constant inspiratory flow and volume-controlled mode with tidal volume (*V*_T_) = 6 ml/kg, minute ventilation (*V*’_E_ = 160 ml/min, inspiratory-to-expiratory ratio = 1:2, fraction of inspired oxygen (FiO_2_) = 0.4, and different levels of PEEP (3, 5.5, 7.5, 9.5, and 11 cmH_2_O) for 1 h. Six rats with ARDS were not mechanically ventilated (nonventilated group) and were used as controls for molecular biology analysis. Lung mechanics, histology, and molecular biology were analyzed.

### Respiratory system mechanics

**Fig. 1:**
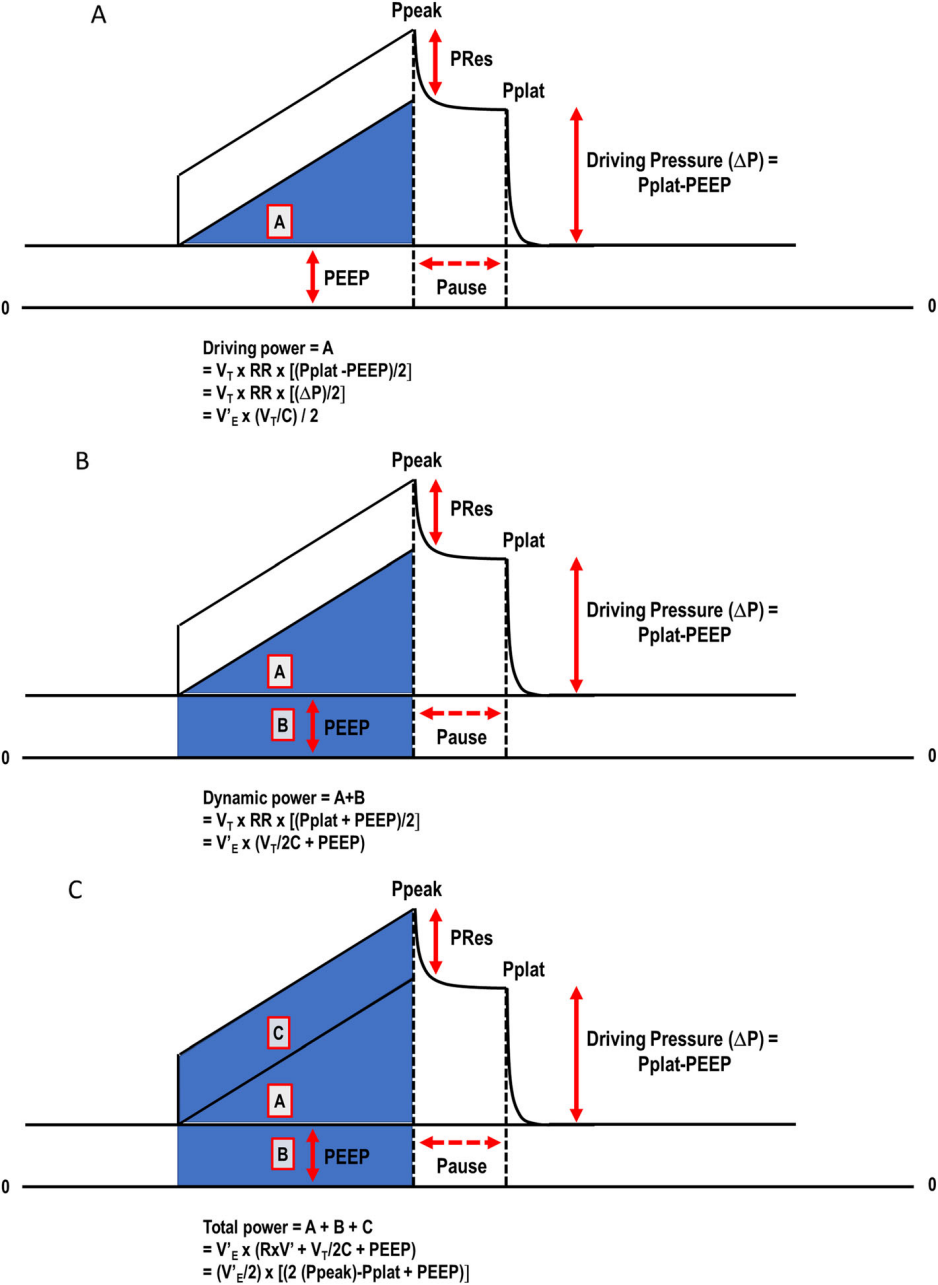
Representative pressure-volume relationship during constant inspiratory flow showing the areas that define the driving power (area A), elastic (dynamic) power (areas A + B), and total power (areas A + B + C). Ppeak, respiratory system peak pressure; PRes, respiratory system resistive pressure; Pplat, respiratory system end-inspiratory (plateau) pressure; ΔP, driving pressure; PEEP, positive end-expiratory pressure; *V*_T_, tidal volume; RR, respiratory rate; *V*’_E_, minute ventilation; C, compliance

Total airway pressure (*P*_app_) is comprised of three pressure elements: flow resistive [airflow (*V*’) × resistance (*R*)], tidal elastic (ΔP), and baseline (PEEP). This relationship is expressed in the simplified “equation of motion”: *P*_app_ = *V*’ × *R* + ΔP + PEEP. When each pressure element is multiplied by the volume displaced from its end-expiratory baseline value, its individual contribution to the total energy of tidal inflation is defined. This relationship among the three energy contributors can be depicted graphically as the pressure-volume areas swept out during inflation (Fig. 1). The PEEP and ΔP areas represent the static and dynamic components of the delivered elastic energy (both energy components are stored at end-inflation and discharged in deflation). During constant inspiratory flow, a commonly used ventilating mode in clinical practice, time becomes an analog of inflation volume, and during passive inflation, pressure-time geometrical areas correspond to the three aforementioned tidal energy components (Fig. 1). Multiplication of these component areas by respiratory rate yields their respective contributions to total power [2, 13]. Driving power, elastic (dynamic) power [the sum of the components relating to driving pressure and PEEP], and total power were computed according to the following formulas: driving power = *V*_T_ × RR × [(Pplat − PEEP)/2], expressed alternatively as *V*’_E_ × (*V*_T_/C)/2 (Fig. 1a), dynamic power = *V*_T_ × RR × [(Pplat + PEEP)/2], or alternatively *V*’_E_ × (*V*_T_/2C + PEEP) (Fig. 1b), and total power = *V*’_E_ × (*R* × *V*’ + *V*_T_/2C + PEEP), or alternatively (*V*’_E_/2) × [2 (*P*_peak_) − *P*_plat_ + PEEP] (Fig. 1c), where *P*peak and *P*plat are respiratory system peak and plateau pressures. At the end of the experiments, lungs were removed for histological and molecular biological analyses.

### Lung histology

At the end of experiment, heparin (1000 IU) was injected in the tail vein. The trachea was clamped at end-expiration, and the lungs were removed *en bloc*. The left lung was frozen in liquid nitrogen. Frozen lungs were fixed in Carnoy’s solution (ethanol/chloroform/acetic acid at a 70:20:10 ratio) at − 70 °C for 24 h. Solutions with progressively increasing concentrations of ethanol at − 20 °C were then substituted for Carnoy’s solution until a 100% ethanol concentration was reached. The tissue was maintained at − 20 °C for 4 h, warmed to 4 °C for 12 h, and then allowed to reach and remain at room temperature for 2 h. After fixation, the tissue was embedded in paraffin. Slices 4 μm thick were obtained by means of a microtome and stained with hematoxylin-eosin.

Lung morphometric analysis was performed using an integrating eyepiece with a coherent system consisting of a grid with 100 points and 50 lines of known length coupled to a conventional light microscope (Olympus BX51, Olympus Latin America, Rio de Janeiro, Brazil). Both dorsal and ventral areas of the lungs were analyzed. The volume fractions of the lung occupied by collapsed alveoli or hyperinflated structures (alveolar ducts, alveolar sacs, or alveoli; maximal chord length in air > 120 μm) were determined by the point-counting technique at a magnification of × 200 across 10 random, noncoincident microscopic fields [14, 15] Briefly, points falling on collapsed and hyperinflated pulmonary areas were counted and divided by the total number of points in each microscopic field.

### Molecular biology analysis of lung tissue

Quantitative real-time reverse transcription polymerase chain reaction (RT-PCR) was performed to measure the expression of markers associated with inflammation [interleukin (IL)-6], alveolar stretch (amphiregulin), and epithelial [club cell protein (CC)-16] and endothelial [intercellular adhesion molecule (ICAM)-1] cell damage in lung tissue [5, 15]. Central slices of right lung were cut, collected in cryotubes, flash-frozen by immersion in liquid nitrogen, and stored at − 80 °C. Total RNA was extracted from frozen tissues using the RNeasy Plus Mini Kit (Qiagen, Hilden, Germany) for the lungs and RNeasy Fibrous Tissue Mini Kit (Qiagen, Hilden, Germany) for the diaphragm, following the manufacturer’s recommendations. The RNA concentration was measured by spectrophotometry in a Nanodrop ND-1000 system. First-strand cDNA was synthesized from total RNA using a Quantitec reverse transcription kit (Qiagen, Hilden, Germany). Relative mRNA levels were measured by SYBR green detection in an ABI 7500 real-time PCR system (Applied Biosystems, Foster City, CA, USA). Samples were measured in triplicate. For each sample, the expression of each gene was normalized to that of the housekeeping gene *36B4* (acidic ribosomal phosphoprotein P0) and expressed as fold change relative to non-ventilated animals, using the 2^−ΔΔ*Ct*^ method, where ΔCt = Ct (reference gene)—Ct (target gene). All analyses were performed by authors who were blinded to group assignment.

### Statistical analysis

Since we used data from a different protocol, the sample size was calculated on the basis of pilot studies which detected differences in IL-6 between PEEP 3 and PEEP 9.5. A sample size of six animals per group would provide the appropriate power (1–*β* = 0.8) to identify significant differences in IL-6 (adjusted *α* = 0.025 for two comparisons), taking into account an effect size *d* = 2.0, a two-sided *t* test, and a sample size ratio of 1 (G^*^Power 3.1.9.2, University of Düsseldorf, Düsseldorf, Germany).

Each variable was tested for normality using the Kolmogorov–Smirnov test. Data are presented as mean ± SD unless otherwise specified. Comparisons among lung functional data were performed using one-way ANOVA with Bonferroni post hoc test among groups. Spearman correlations of driving power, dynamic power, total power, expression of biological markers, alveolar collapse, and hyperinflation were calculated using PEEP from 3 to 9.5 and 3 to 11 cmH_2_O since significant changes in lung mechanics, morphology, and molecular biology were observed between 9.5 and 11 cmH_2_O PEEP. All tests were performed in GraphPad Prism v6.07 (GraphPad Software, USA). Significance was established at *p* value less than 0.05.

## Results

All animals survived to the end of the experiment and were kept hemodynamically stable during 1 h of mechanical ventilation. At the final time, no statistically significant differences among groups were observed in the *V*_T_ and RR. As expected, both *P*peak and *P*plat increased progressively over the tested range of PEEP (Table 1).

**Table 1:** Respiratory variables used to calculate driving power, dynamic power, and total power at each PEEP level, at the end of the experiment

Variables	PEEP (cmH_**2**_O)				
	3	5.5	7.5	9.5	11
***V***_**T**_**(ml)**	2.2 ± 0.2	2.2 ± 0.0	2.1 ± 0.1	2.0 ± 0.1	2.2 ± 0.3
**RR (bpm)**	75 ± 8	74 ± 2	75 ± 5	81 ± 4	75 ± 9
**Ppeak (cmH**_**2**_**O)**	12.2 ± 1.8	15.1 ± 0.7	17.4 ± 1.2*	22.3 ± 2.4*^#&^	30.1 ± 4.3*^#&^**
**Pplat (cmH**_**2**_**O)**	9.7 ± 1.4	12.9 ± 0.6*	15.1 ± 0.8*^#^	18.3 ± 2.5*^#&^	24.2 ± 3.1*^#&^**
**ΔP (cmH**_**2**_**O)**	8.4 ± 1.4	8.8 ± 0.6	9.5 ± 0.4	10.5 ± 2.3	14.7 ± 2.9*^#&^**

Values are means + SD of 6 animals/group

One-way ANOVA followed by the Bonferroni post hoc test was performed

*V*_*T*_ tidal volume, *RR* respiratory rate, *Ppeak* respiratory system peak pressure, *Pplat* respiratory system end-inspiratory (plateau) pressure, *ΔP* driving pressure, *PEEP* positive end-expiratory pressure

*Significantly different from PEEP 3 (*p* < 0.05)

^#^Significantly different from PEEP 5.5 (*p* < 0.05)

^&^Significantly different from PEEP 7.5 (*p* < 0.05)

**Significantly different from PEEP 9.5 (*p* < 0.05)

**Fig. 2:**
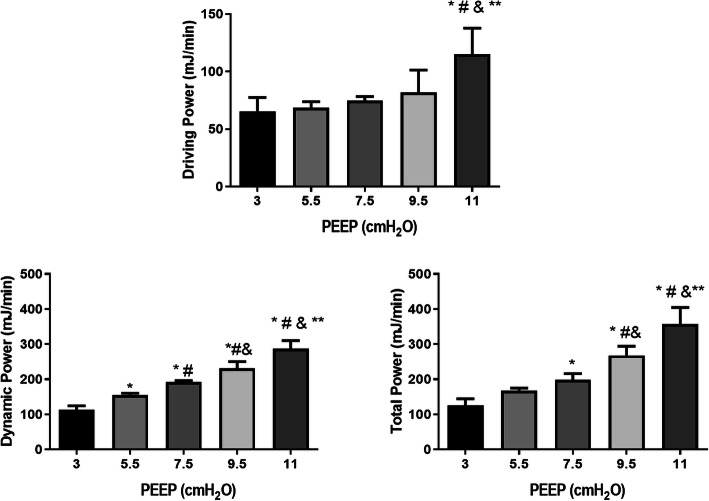
Driving power, dynamic power, and total power applied at different levels of PEEP, at the end of the experiment. All animals were mechanically ventilated with *V*_T_ = 6 ml/kg for 1 h. Values are mean + SD of 6 animals/group. Asterisk indicates significantly different from PEEP 3 (*p* < 0.05); number sign indicates significantly different from PEEP 5.5 (*p* < 0.05); ampersand indicates significantly different from PEEP 7.5 (*p* < 0.05); double asterisk indicates significantly different from PEEP 9.5 (*p* < 0.05). One-way ANOVA followed by the Bonferroni post hoc test was performed

Unlike elastic pressure and dynamic power, driving pressure (Table 1) and driving power were higher only at PEEP 11 cmH_2_O compared with other levels of PEEP (Fig. 2). Conversely, elastic (dynamic) power increased significantly and progressively across the range of PEEP (Fig. 2).

**Fig. 3:**
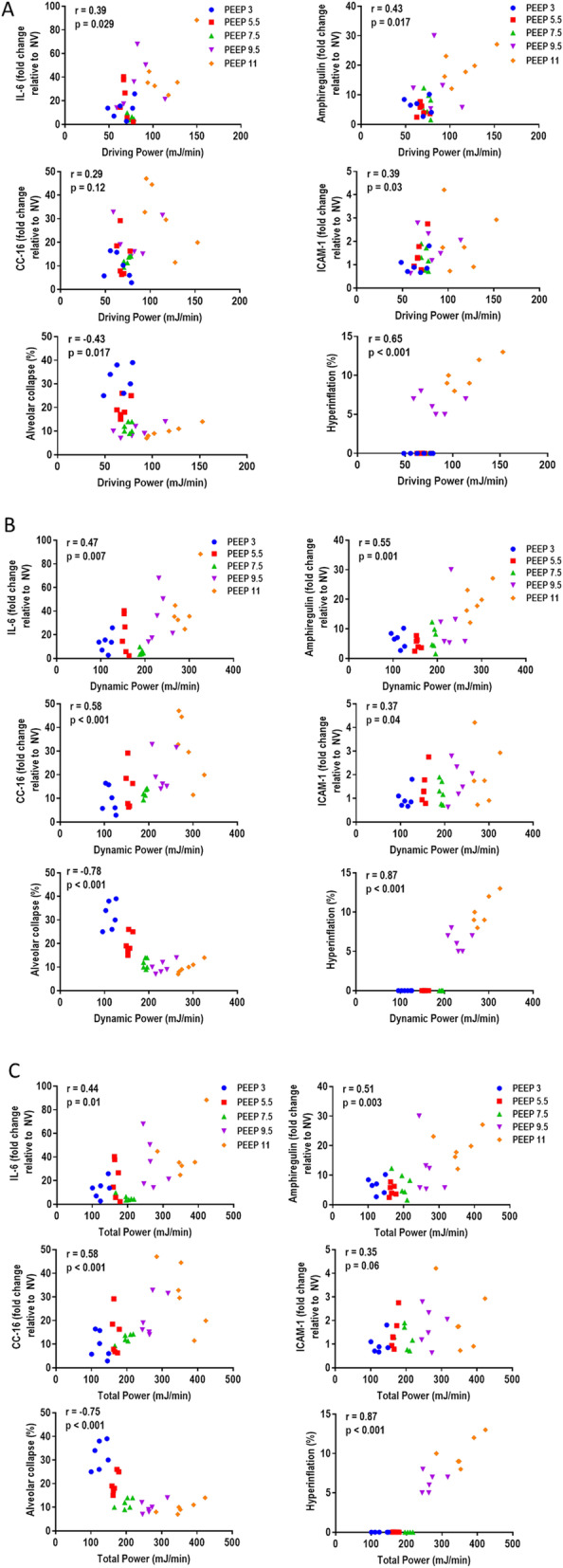
Spearman correlations of driving power (**a**), dynamic power (**b**), and total power (**c**) with gene expressions of IL (interleukin)-6, amphiregulin, CC (club cell protein)-16, and ICAM (intercellular adhesion molecule)-1, as well as fractional areas of alveolar collapse and hyperinflation from PEEP 3 to PEEP 11 cmH_2_O. *r* = correlation coefficient with respective *p* value

Over a wide range of PEEP, dynamic power correlated more closely with injury indicators than did driving power. Correlation analyses of mechanical, biological, and morphological data among all groups showed the following: from PEEP 3 to PEEP 11 cmH_2_O: (1) driving power correlated with all injury indicators except for CC-16 expression (Fig. 3a), (2) dynamic power correlated with all injury indicators (Fig. 3b), and (3) total power correlated with all injury indicators except for ICAM-1 expression (Fig. 3c); from PEEP 3 to PEEP 9.5 cmH_2_O: (1) no correlations were observed between driving power, biomarkers, and lung histology (Fig. 1a, Additional file 1, Figure S1) and (2) elastic (dynamic) power and total power were correlated with CC-16 expression (*p* = 0.03, *p* = 0.02, respectively), alveolar collapse (*p* < 0.001 for both), and hyperinflation (*p* < 0.001 for both) (Fig. 1b and c, Additional file 1, Figure S1).

## Discussion

Our data clearly demonstrate that elastic power (dynamic power) parallels total power as a VILI risk indicator when PEEP is the primary tidal variable used to influence cumulative stress exposure. In contrast, under these conditions of fixed tidal volume and rising PEEP, driving power (the driving pressure × respiratory rate product) did not bear significant correlations to biomarkers associated with inflammation, alveolar stretch, and epithelial and endothelial cell damage as well as alveolar collapse and hyperinflation over much of their ranges. Only at the highest level of PEEP did driving power increase significantly, causing the related correlations with injury to strengthen. These findings, therefore, strongly suggest a “threshold” effect for repetitive stress and strain, with total and elastic power values < 100 mJ/min inconsistently related to lung histologic or biomarker indications of damage in this model.

Airway driving pressure and static end-inspiratory (“plateau”) pressures continue to serve as simple bedside indicators of VILI vulnerability [16]. It seems rather clear, however, that on a biophysical level, understanding their causal connections to tissue stress and strain is incomplete [17–21]. Because energy input is required to inflict the micro-wounding stimulus, the product of applied pressure and the resulting volume holds more persuasive appeal. It follows that the intensity (power) and cumulative number of such high energy tidal cycles also must be considered [2].

Even as attempts to explain VILI shifted from the volume and pressures of the individual tidal cycle toward the unifying concept of power, uncertainty has remained regarding whether the measurement of total power itself needs further modification to improve its predictive precision. In fact, because the flow-resistive and PEEP-related components of tidal pressure are less intuitively related to tissue damage than is the driving pressure component, it is conceivable that the product of driving pressure and respiratory rate provides all needed information. An intricate statistical analysis of data from well-done randomized clinical trials indicated that driving pressure alone was the key variable, independent of the associated PEEP and plateau pressures [16]. Yet, because repeated excessive strain is the likely initiator of tissue damage from ventilation, it stands to reason that surpassing a threshold value for tidal strain is a prerequisite for driving power to prove damaging. These data support that interpretation.

Our analysis, a completely new exploration of unpublished granular data demonstrates that the repeated application of the driving pressure itself is not the key to VILI in this experimental setting; with tidal volume regulated at 6 ml/kg, driving power was clearly not as tightly correlated with biomarker and histologic expressions of VILI as the PEEP-inclusive dynamic power. Indeed, across a wide range of driving power, there was no consistent relationship of driving power and injury. Only at the highest level of PEEP-driven power did a weak correlation emerge, as both driving pressure and plateau pressures (driving pressure plus PEEP) rose significantly over their baseline values. In contrast, the dynamic power, which is the product of respiratory rate and the sum of PEEP and driving pressure, bore a strong relationship to VILI. Taken together, such data suggest that a threshold value of stretching pressure must be crossed before injury begins, and in this context, PEEP serves as a platform upon which the driving power can exert its damaging influence. To our knowledge, these results offer the first clear demonstration by measures that include biochemical and molecular methods that PEEP in excess of that which overcomes widespread atelectasis can induce VILI. These findings complement those of Collino and colleagues in large animals that indicated the contribution of high PEEP to VILI [11].

Total power was also well correlated with injury, but the addition of the flow-resistive power component to dynamic power improved that correlation only marginally. Thus, rising PEEP is not innocuous, as it may allow maximal tissue stresses to breach a damage threshold, beyond which repeated high energy cycles hold potential for injury.

### Limitations

Even though this rodent model of ARDS clearly is not directly and quantitatively comparable to other animal models or to clinically encountered ARDS, its potential value for practice is in elucidating the mechanisms that underlie both. On an absolute scale, the applied pressures may seem modest by large animal or human standards but actually are quite high—even life-threatening—for the small animal lung [22]. As reflected in the evidence we present regarding hyperinflation and atelectasis, these experiments were conducted over a moderate to high range of stress and strain. Indeed, PEEP levels higher than the highest one tested could not be sustained without catastrophic hemodynamic compromise. Additionally, the PEEP levels used in the present study (3–11 cmH_2_O) are often applied in rats and are approximately equivalent to double those in humans (6–22 cmH_2_O).

This is not a reanalysis of previously published data, but a new analysis of experiments performed in our laboratory which were designed to answer a different question than the one we asked here; we did not test each of the three pressure components of tidal energy as independent variables nor extend data collection over a period longer than 1 h. Because PEEP was the primary driver of tidal energy and of power, resistance and tidal volume were not changed. Nonetheless, both driving pressure and plateau pressure, the key variables of interest, varied over an extended span, and the strong suggestion of an injury threshold for these pressures was uncovered.

## Conclusions

From these data, we conclude that dynamic power, not driving power, is the key promoter of VILI. Moreover, the hazards from driving pressure and driving power are conditioned by the requirement to pass a tidal stress threshold. When estimating VILI hazard from repeated mechanical strains, therefore, PEEP must not be disregarded as a major contributor to injury risk and target for modification.

## Supplementary information

**Additional file 1.** A Word file showing Spearman correlations of driving power, dynamic power and total power with gene expressions of biomarkers and lung morphometry (from PEEP 3 to PEEP 9.5 cmH_2_O).

## Data Availability

The datasets used and/or analyzed during the present study are available from the corresponding author on reasonable request.

## Abbreviations

ARDS: Acute respiratory distress syndrome
C: Compliance
ΔP: Driving pressure
FiO_2_: Fraction of inspired oxygen
IL: Interleukin
CC-16: Club cell protein 16,
ICAM-1: Intercellular adhesion molecule-1
PEEP: Positive end-expiratory pressure
Ppeak: Respiratory system peak pressure
Pplat: Respiratory system end-inspiratory (plateau) pressure
RR: Respiratory rate
RT-PCR: Quantitative real-time reverse transcription polymerase chain reaction
*V*’_E_: Minute ventilation
VILI: Ventilator-induced lung injury
*V*_T_: Tidal volume
