# Supplementary material for: Elastic power but not driving power is the key promoter of ventilator-induced lung injury in experimental acute respiratory distress syndrome
Source: Crit Care. 2020 Jun 3;24:284. doi: 10.1186/s13054-020-03011-4 (PMC7271482; doi:10.1186/s13054-020-03011-4)
Supplement: Supplementary file 1 — Additional file 1. A Word file showing Spearman correlations of driving power, dynamic power and total power with gene expressions of biomarkers and lung morphometry (from PEEP 3 to PEEP 9.5 cmH2O). [file 13054_2020_3011_MOESM1_ESM.docx]

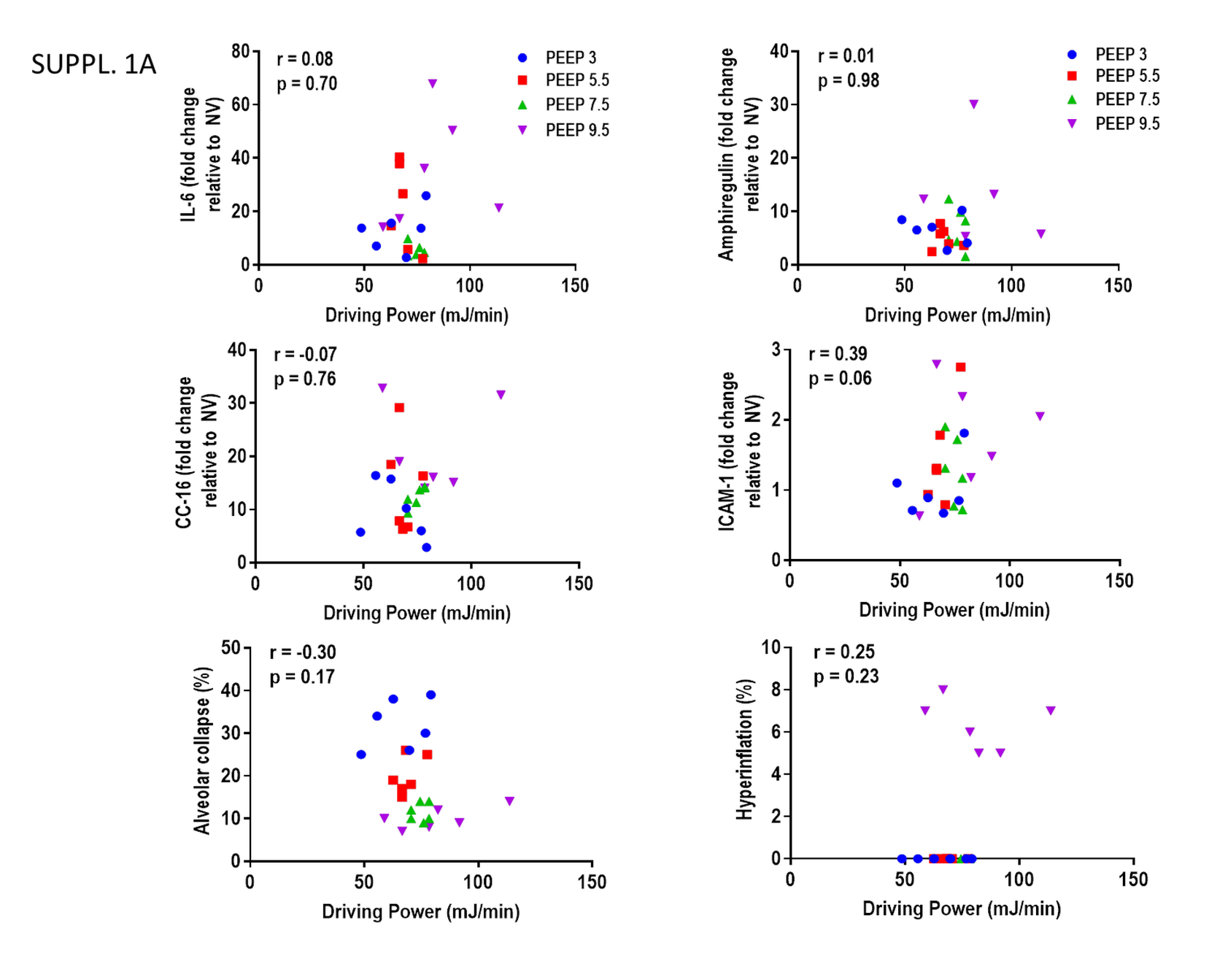


**A**


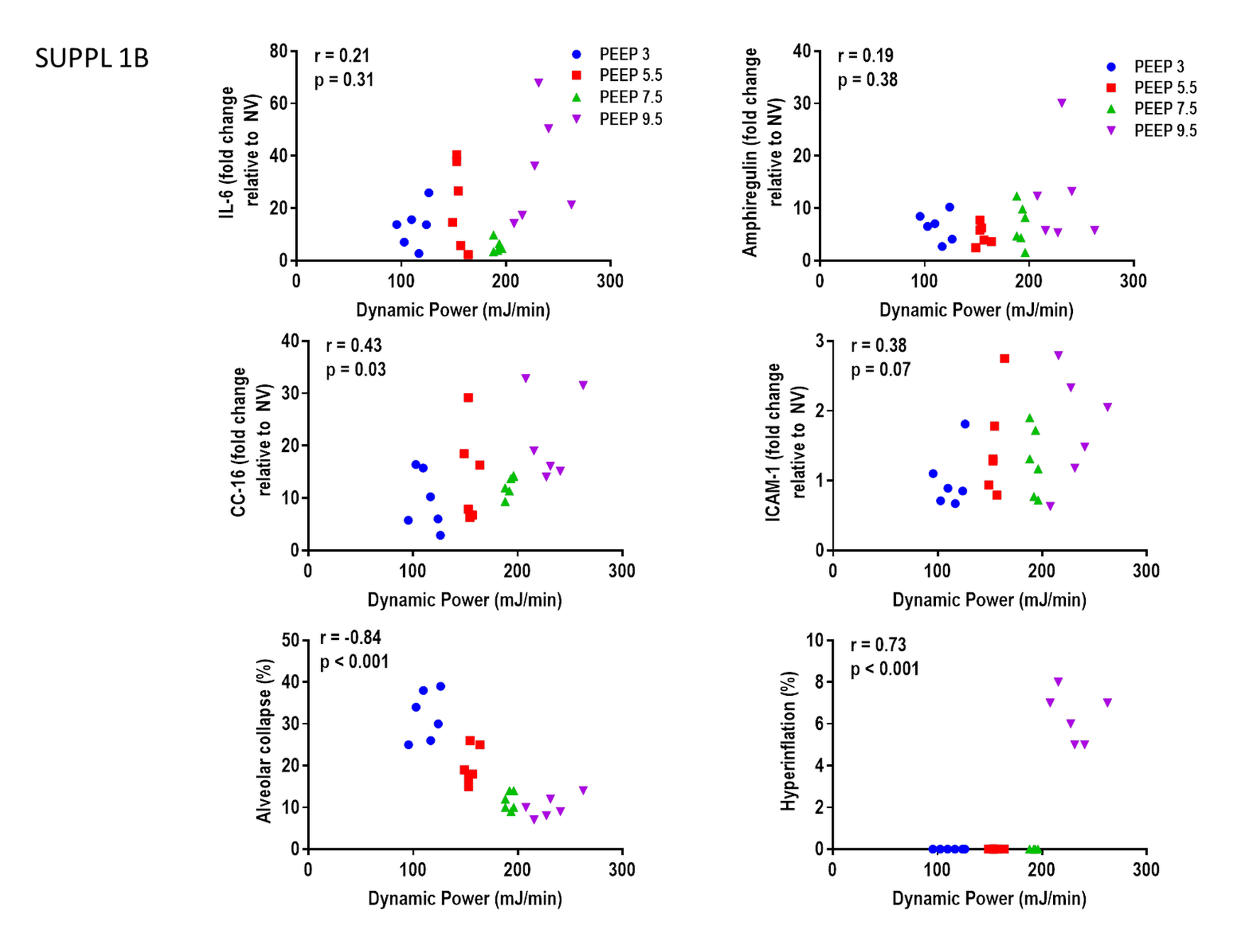


**B**


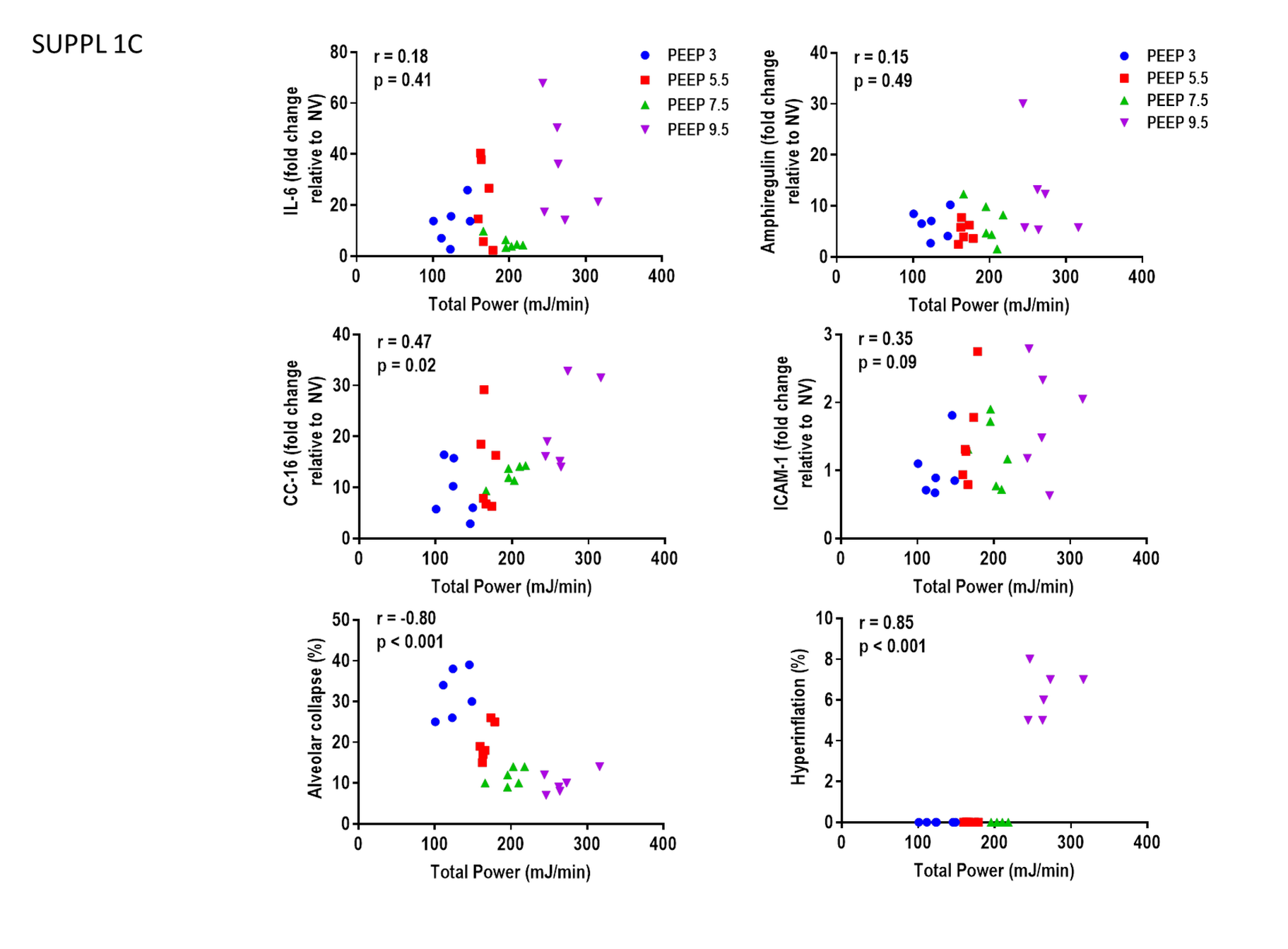


**C**

**Figure S1.** Spearman correlations of driving power (A), dynamic power (B) and total power (C) with gene expressions of IL (interleukin)-6, amphiregulin, CC (club cell protein)-16, ICAM (Intercellular Adhesion Molecule)-1, as well as fractional areas of alveolar collapse and hyperinflation from PEEP 3 to PEEP 9.5 cmH_2_O. r = correlation coefficient with respective p value.
